# Women’s experiences of the journey to chronic widespread pain: a qualitative study

**DOI:** 10.1186/s12891-020-03442-8

**Published:** 2020-06-30

**Authors:** Miriam Svensson, Ingrid Larsson, Katarina Aili

**Affiliations:** 1 School of Health and Welfare, Halmstad University, P.O. Box 823, S-301 18 Halmstad, Sweden; 2 Department of Health and Caring Sciences, Faculty of Health and Life Sciences, Linnaeus University, Kalmar, Sweden; 3 Spenshult Research and Development Centre, Halmstad, Sweden; 4 Department of Clinical Sciences, Section of Rheumatology, Lund University, Lund, Sweden; 5 Unit of occupational medicine, Institute of Environmental Medicine, Karolinska Institutet, Stockholm, Sweden

**Keywords:** Chronic widespread pain, Interviews, Pain development, Pain journey, Patient experiences, Qualitative study, Qualitative content analysis, Women

## Abstract

**Background:**

Chronic widespread pain (CWP) is a musculoskeletal disorder that affects approximately 10% of the population. It is more common in women than in men. It is important to understand how CWP develops and how it is maintained in order to prevent poor pain prognosis. Long term studies have shown that a mere part improves over time or fluctuates in their CWP condition. Female gender is one of the factors associated with persistence of CWP, suggesting men and women may experience their journey to CWP differently. The aim of the study was to explore women’s experiences of the journey to CWP.

**Methods:**

19 women between 45 and 67 years of age who had not reported CWP in the EPIPAIN survey in 1995, but reported CWP in 2016, participated in the study. Data was collected through individual interviews, where open-ended questions were used to explore the women’s experiences of their pain journey. The interviews were analyzed with a manifest qualitative content analysis.

**Results:**

The women described their journey to CWP in terms of triggering, aggravating, and consolidating factors, from which three different categories emerged. *Experiencing that environmental circumstances affect the pain journey* refers to factors outside the women’s immediate control, which appeared as unmanageable work-related demands, lack of social support, unfavorable physical environments, and traumatic events. *Experiencing that lifestyle affects the pain journey* refers to events that are consciously or unconsciously carried out by the women, including different levels of physical efforts and unfavorable behaviors. *Experiencing that personal attributes affect the pain journey* refers to the women’s characteristics in terms of an anxious state of mind and adverse biological impact.

**Conclusions:**

The women experienced that environmental circumstances, lifestyle, and personal attributes affected their CWP. How these adversities influenced the pain journey varied among the women. These findings show that women are conscious of the complexity of the condition and can describe the broad context of their pain journey. This study confirms the complexity of pain progress and highlights the individual’s awareness of this complexity, which is important to consider when introducing interventions, and when expecting compliance to interventions.

## Background

Chronic widespread pain (CWP) is a musculoskeletal disorder that affects approximately one in ten people in the general population, with an estimated overall prevalence of 11.2% in women and 7.2% in men [1]. It is also more common in middle-aged groups [2]. Living with CWP affects both the person’s health and lifestyle at the same time as entailing huge costs for society [1].

Musculoskeletal pain can be classified by the number and localization of pain-sites, where CWP refers to pain lasting longer than 3 months, with the pain being on the left and right side of the body, above and below the waist and in the axial skeleton [3]. Compared to localized pain, CWP has a larger impact on health, as the number of pain-sites has shown to have an almost linear relationship with a reduced functional ability [4] and an inverse linear relationship with general health, sleep quality and mental health [5]. The etiology remains undefined but it is known that CWP has a number of different causes. A widely accepted model that so far has seemed to yield the most promising explanation for the etiology of CWP, is the biopsychosocial model. It views physical illness, such as CWP, as a result of a complex interplay between physiological, psychological, and social determinants [6, 7]. For example, factors can affect the susceptibility, initiation, maintenance, and aggravation of the pain, while a negative mood can influence treatment motivation and compliance with treatment recommendations [6, 7]. As CWP integrates with several complex factors like these, it is challenging to treat people with this condition [8].

To prevent poor pain prognosis and development of CWP, and to make treatments more effective, it is important to understand how pain develops and how it is maintained. Research related to this has focused on biographical disruption [9, 10]. The concept encompasses the way that chronic illness can challenge or redefine a person’s sense of identity and social relationships. First, symptoms are excused by other factors. When these explanations are no longer sufficient, sufferers begin to search for a diagnosis. As it is often difficult to receive a medical diagnosis and to be given a cure or a full explanation of the causes of the illness, sufferers search for complementary information about their own lives that can help them understand the cause and meaning of their illness [11]. Even though the concept of biographical disruption has been criticized for being insufficient in acknowledging the importance of examining the context of the lives in which the disruption occurs [9, 12], it is suggested to be an important concept in the context of CWP when taking this limitation into account [9]. Research namely shows that persons with CWP attempt to give meaning to their present pain experience and their whole life, by using different frameworks when talking about the onset of their pain; either focusing on triggering incidents and/or on predisposing factors [9]. However, qualitative research exploring factors like these that could help gain a better understanding of the pain journey, are lacking.

Furthermore, although CWP is considered a chronic condition, long-term studies including more than two time-points for follow-up have shown that the condition can only be regarded as persistent over time for approximately 50% [13]. This suggests that when identifying a group of individuals with CWP at a specific time point, about half of them belong to a group with CWP that will fluctuate between regional pain and CWP, or no chronic pain. Although more research is needed to better understand CWP over a life span, it is possible that fluctuating CWP and persistent CWP may represent different dimensions of the condition. One of the factors predicting persistence of CWP over time is female gender [14, 15]. It is, thereby, possible that men and women to some extent experience their pain journey over a life span differently, which should be considered when including men and women in the same study. Including women only, and men only, in qualitative studies describing experiences of developing CWP would allow for the possibility that the experiences to some extent may be influenced by gender. Further, the prevalence of CWP differs between age groups. Among women, the prevalence of chronic pain seems to increase the most in age groups between 35 years old and 50 years old [2], which implies that many of the women who develop CWP are experiencing their pain journey during these years. No studies have been found that focus on experiences during this pain journey.

As argued above, “a chronic pain condition” is a very broad description of a condition that represents a heterogeneous group of individuals suffering from rather different conditions. In order to learn more about the individuals who develop persistent CWP, a more strict approach when selecting study participants should be applied. It was, thereby, under the scope of this study to explore different experiences of persistent CWP among individuals who ought to have gone through similar pain journeys. Therefore, only middle-aged women who had developed CWP during their adult life were of interest in this study. The aim of this study was thus to explore women’s experiences of the journey to CWP.

## Methods

### Study design

This is an explorative study, where a qualitative content analysis with an inductive approach was used.

### Participants

The participants in this study were selected from responders to the EPIPAIN study [16]; a prospective population study initiated in 1995, which was designed to investigate prevalence and risk factors for long term musculoskeletal pain in a population in southern Sweden. In the EPIPAIN study, a representative sample of 3928 people was selected by choosing every 18th man and woman from an official computerized population register ordered by date of birth including all 70,704 individuals between 20 and 74 years of age, living in two municipalities in southern Sweden. Out of the 3928 invited individuals, a total of 2425 individuals responded to the postal survey in 1995 and were sent follow-up questionnaires in 1998, 2003, 2007, and 2016 [16]. To find participants for this study, information from the EPIPAIN study about the prevalence of CWP among women in 1995 and 2016 was used. Women, older than 40, who had not reported CWP in 1995, but reported CWP in 2016, were of interest for this study [2]. A total of 92 women met this criterion. A purposeful selection, considering age, civil status, educational level, employment, and pain sites (Table 1) was made in order to get variation among the participants [17]. Since qualitative content analysis seeks after variation in content and multiplicity and to ensure enough data to cover significant variations it was decided to include 20 women in the study [18]. The women were initially approached by mail and asked to participate. If someone declined to participate or did not respond after two reminders, a representative woman of the same age was chosen instead. A total of 43 women received information about the study and were given the opportunity to participate, 19 of whom participated in the study, 9 declined to participate and 15 did not answer. The women who declined to participate gave no further reasons that explained their decision.

**Table 1:** Details of the participants

Participating women	*N* = 19
Age (years)	
Median	57
Range	45–67
Civil status (n)	
Married/cohabitant	14
Single	5
Educational level (n)	
Primary school	2
Secondary school	12
College/University	5
Employment (n)	
Working full or part time	13
Sick-listed	3
Retired	3
Pain sites	
Median	8
Range	4–16

At the time for the interview, the included women had experienced CWP for a different amount of time, since the criterion for being included was that the onset of CWP should have occurred at some point between 1995 and 2016. In 1998, two women reported CWP, in 2003 seven women reported CWP, in 2007 ten women reported CWP, and in 2016 all 19 women reported CWP. All women except one reported CWP persistently from the time for onset, and onwards. The woman who fluctuated in the condition reported CWP in 2003, but not in 2007, then again in 2016.

### Data collection

Data was collected through individual interviews, where each participant was interviewed on one occasion in a secluded room at a Research and Development Centre. The interviews took place between May 2017 and November 2017 and were either held by the author IL or KA, who had not previously met the participants. Each interview lasted between 25 and 96 min (median 59 min, total 18 h and 35 min) and was audio-recorded and transcribed verbatim.

Two pilot interviews were first conducted to test the understanding of the questions, and since revision was not required, these interviews were included in the analysis. Each interview began with the interviewer clarifying the aim of the study, its voluntary nature, and that the women could withdraw from the interview at any time. Identical open-ended questions were used to ensure that similar data was gathered from all participants [19]: *“What does pain mean to you?”*, *“How do you experience your pain?”*, *“Can you describe how your pain has developed over the last 20 years?”*, *“How did your pain change over time?”* and “*Have you experienced any important events that have influenced the development of your pain?”*. Follow-up questions such as: *“Please tell me more”*, *“How do you mean?”* and *“What do you have in mind when you say …*? *”*, were also used to encourage the women to give more detailed responses [19].

### Data analysis

Data were analyzed by manifest qualitative content analysis with an inductive approach, in accordance with Graneheim et al. [18, 20]. The focus of the analysis was on identifying similarities and differences in the content of the text, describing variations, and increasing the understanding of the phenomenon chosen. The intention was to remain close to the transcribed text and preserve the contextual meaning of the women’s experiences, by moving between the whole and the parts of the transcribed text throughout the analysis [20]. Since the study had an inductive approach, the purpose was to gain a more abstract and general theoretical understanding based on the concrete and specific data [18].

The analysis started with selecting *units of analysis*, which in this case consisted of all 19 interviews. The transcribed interviews were thereafter read through several times in order to gain a sense of the whole. A total of 166 *meaning units* representing constellations of words from central meanings relevant to the aim of the study, were then identified and extracted. These meaning units were *condensed* to shorten the text, without the content being affected. The condensed meaning units were *abstracted*, or in other words, labelled with *codes* that briefly described the content. Codes of similar content were grouped into eight *subcategories*. For example, the codes ´heredity triggers the pain` and ´age aggravates the pain` were grouped into the subcategory ´adverse biological impact`. The subcategories were then grouped into three *categories*, which reflected the core subject matter of the interviews and formed the manifest content [20].

## Results

The women experienced that environmental circumstances, lifestyle, and personal attributes affected their journey to CWP. Environmental circumstances included events that the women could not influence directly themselves, while lifestyle referred to events that consciously or unconsciously were carried out by the women. Personal attributes were characteristics of the women, congenital as well as acquired (Table 2). The women expressed the pain journey in terms of triggering, aggravating, and consolidating factors. Triggering factors reflected experiences that led to the onset of CWP. Aggravating factors included experiences that increased the pain while consolidating factors were experiences that made the pain constant.

**Table 2:** Example of the qualitative content analysis based on the women’s experiences of the journey to CWP

Meaning unit	Condensed meaning unit	Code	Subcategory	Category
The pain I have in my hips and shoulders and so on, them I have got gradually because of my job … // My work is very physically demanding … [3]	The pain arose because of my job, which is physically demanding.	Physically demanding work triggers the pain	Unmanageable work-related demands	Experiencing that environmental circumstances affect the pain journey
“… if you hadn’t believed what I said and I had left here with a feeling of no I could see that she didn’t believe me at all, and if you had been a doctor, then that increases my pain and I become sad and everything is built up inside” [17]	Everything is built up from within and if someone doesn’t believe me, I become sad and then the pain is aggravated.	Mistrust aggravates the pain	Lack of social support	
“It’s the worst for me in the summer when it’s hot, then it feels as though my joints are burning” [12]	The pain is at its worst in the summer when it is hot.	Hot weather aggravates the pain	Unfavorable physical environments	
“… then I lost my husband two and a half years ago, and then I realized that I was really depressed because it was difficult but then there is the mental stress as I felt like shit and had a lot of pain … so it’s not just heavy work it’s the mental aspect that affects the pain also” [6]	When my husband passed away, I felt mentally ill and stressed, which aggravated the pain.	Heartache aggravates the pain	Traumatic events	
(reason for back problems) “I’ve been sitting down too much .. Sitting down too much and not training enough.” [4]	The pain arose because I was too physically inactive.	Inactivity triggers the pain	Different levels of physical efforts	Experiencing that lifestyle affects the pain journey
“… and I think that it’s because I haven’t dealt with the pain, instead I’ve just thought that it will pass, it will get better …” [6]	The pain has remained as I haven’t dealt with it.	Neglecting consolidates the pain	Unfavorable behavior	
“Then if I get more worried or like this when it, yes if I just think about the children and things like that, if I say the grandchildren and when it’s something with them and I get stressed, then I get more tense and then feel more pain in my shoulders and my back …” [11]	When I worry about people dear to me the pain is aggravated.	Worries aggravate the pain	Anxious state of mind	Experiencing that personal attributes affect the pain journey
“… it started sometime in my 40s, because then I identified myself with older colleagues who were in pain then … I told them that, aha, now it starts on me and then it was probably a knee or something that wasn’t working properly.” [1]	The pain started in my 40s, when I began to identify myself with older colleagues who were in pain.	Age triggers the pain	Adverse biological impact	

### Experiencing that environmental circumstances affect the pain journey

This category referred both to physical and social environments and included unmanageable work-related demands, lack of social support, unfavorable physical environments, and traumatic events that affect the pain journey. More specifically, these adversities represented external factors that were beyond the women’s immediate control.

#### Unmanageable work-related demands

Unmanageable work-related demands included the impact of work, both physically and mentally, on the women’s pain journey. Since the women were not responsible for the work demands and were economically dependent on their employment, these factors were seen as external circumstances. Work-related adversities in relation to CWP had triggering, aggravating, and consolidating effects. The varying level of physical demands in the work affected the pain, where some had developed pain from inactive work and others from physically demanding work. The work led to different types of physical damage, such as a repetitive strain injury and torticollis, which in return aggravated the pain. Stress and pressure from their employers also had a negative impact on the pain journey along with the physical demands. One woman described work-related demands as:

“The pain that I have in my hips and shoulders, it’s come on gradually due to my work..// My work is very physically demanding ..” [3]

Furthermore, a difficult labor market, in general, was experienced as an aggravating factor for CWP, as well as working more than the pain allowed. A different experience was that the pain was equally bad regardless of whether the person was at work or off duty, while another feeling was that the pain sometimes aggravated when not being on duty. One woman explained this as:

“… sometimes I actually feel more pain (when I’m off), but I think that is maybe because I relax, then I feel it even more …” [18].

#### Lack of social support

Lack of social support included how the women were treated by others and how they were affected by social norms, which were difficulties the women had no control over. The women experienced feelings of distrust and not being understood, especially by health care professionals and insurance companies, which for some of the women had either triggering or aggravating effects on CWP. In a worst-case scenario, the women were not offered any treatment at all. One woman expressed the mistrust from her social surrounding in terms of:

“… if you hadn’t believed what I said and I had left here with a feeling of no I could see that she didn’t believe me at all, and if you had been a doctor, then that increases my pain and I become sad and everything is built up inside” [17].

Social norms that concerned how women were expected to behave also had an impact on the pain journey. The norms created a feeling of having to oblige everyone, which impaired the women’s ability to take care of themselves and thus aggravated the pain. One woman stated:

“… as a woman one thinks that I’m going to manage this, I’m going to take care of the children, I’m going to take care of our home and everything carries on and one just doesn’t think of oneself. // One has to be there and lend a hand and be obliging for everybody, as it were.” [6]

#### Unfavorable physical environments

This subcategory exemplified how both natural and human-made surroundings, which could be touched or seen, affected the women’s pain journey. An aggravating factor related to this was the weather. There were, however, variations in experiences concerning weather conditions. Some of the women felt more pain when it was cold, rainy, or windy, while others felt more pain when there was hot weather. Furthermore, the surface the women walked or stood on was also experienced as influencing the pain journey, for example, walking on hard floors had an aggravating effect on CWP. One woman expressed the unfavorable physical environment as:

“It’s the worst for me in the summer when it’s hot, then it feels as though my joints are burning” [12].

#### Traumatic events

Some of the women had experienced physical traumas, such as sports injuries or whiplash injuries, while others had experienced psychological traumas in terms of heartache or a difficult childhood. The common denominator for these experiences was, however, that they occurred due to circumstances beyond the women’s control and that they had a triggering, aggravating, or consolidating effect on CWP. One woman described how a trauma increased her CWP:

“… then I lost my husband two and a half years ago, and then I realized that I was really depressed because it was difficult but then there is the mental stress as I felt like shit and had a lot of pain … so it’s not just heavy work it’s the mental aspect that affects the pain also” [6].

### Experiencing that lifestyle affects the pain journey

This category was related to the women’s lifestyle and incorporated different levels of physical efforts and unfavorable behaviors that affected the pain journey due to either conscious or unconscious actions that the women carried out. These difficulties were thus factors the women were able to control.

#### Different levels of physical efforts

Different levels of physical efforts were factors related to the women’s physical state, which they could control. Some experienced physical activities as triggering and aggravating factors of CWP. There was, however, a variation in the intensity and duration of these physical activities, both among the women and from day to day. Some of the women perceived that they were more afraid of physical activities causing pain than others. Recurring experiences were, however, those where more physically demanding actions, such as working out at the gym, skiing, dancing, or running, affected the pain journey. In some cases, however, everyday activities such as walking, carrying grocery bags, cleaning, standing up for a longer time, having sex, or gardening were enough to affect the pain in a negative way. On the other hand, some of the women experienced that being inactive also triggered or aggravated the pain. One woman expressed the impact of physical efforts on CWP as:

“I’ve been sitting down too much .. Sitting down too much and not training enough.” [4]

Other factors that could be affected by physical activity, such as having a bad posture, tension, a low oxygen uptake capacity, or weight gain, were experienced as having an aggravating effect on CWP. Some of the women also experienced that physical effort, such as being pregnant, aggravated the pain. One woman said:

“Yes, it is. I notice that when I feel tense then it (the pain) is worse ..” [18]

#### Unfavorable behavior

This subcategory referred to actions based on the women’s behavior. A triggering, aggravating, and consolidating factor related to CWP was the action to override the needs related to pain, which either occurred consciously or unconsciously. More specifically, these women ignored their physical condition and continued with activities that affected the pain negatively. Other behavioral factors included choices the women could make on their own. These concerned; drinking alcohol, taking medicine, walking barefoot, and wearing uncomfortable shoes, which all had an aggravating effect on CWP. One woman explained behavioral factors in terms of:

“… and I think that it’s because I haven’t dealt with the pain, instead I’ve just thought that it will pass, it will get better …” [6].

### Experiencing that personal attributes affect the pain journey

This category described the personal characteristics of the women, congenital as well as acquired, where an anxious state of mind and an adverse biological impact affected the pain journey. This entailed who the women were and how their personal attributes were conveyed, which made them vulnerable.

#### Anxious state of mind

Having an anxious state of mind was a psychological vulnerability that was frequently considered and said to have both triggering and aggravating effects on CWP. Mental illness was referred to here, and some of the women spoke about it in general terms, while others gave more detailed definitions. Being worried about their financial situation or other things in everyday life, feeling depressed, tired, and exhausted were talked of as having negative effects on CWP. How the women reacted to other people’s problems also had an impact on the pain journey. Some of them worried a great deal about others, were afraid of conflicts, and prioritized others before themselves, which triggered and aggravated the pain. One woman described this as:

“Then if I get more worried or like this when it, yes if I just think about the children and things like that, if I say the grandchildren and when it’s something with them and I get stressed, then I get more tense and then feel more pain in my shoulders and my back …” [11].

Another experience was the feeling of being afraid of the pain, which led to a more nervous state of mind and caused tension, which in return aggravated the pain. Similarly, not understanding the pain, and thus believing that it was a psychological and not a physical complaint, aggravated the pain. One woman reported:

“… thus it’s a physical pain, that’s not something in one’s head. It took such a long time for me to understand that it couldn’t, that I couldn’t actually affect it mentally. Just that it was like stomach pains, and after that I’ve sort of learnt to live with them” [3].

#### Adverse biological impact

Adverse biological impact referred to somatic vulnerabilities, where some of the women felt that heredity had a triggering factor of CWP. Having relatives who also suffered from pain was expressed as an explanation for the pain. Aging or just simply feeling old were similarly experienced as having a triggering, but also an aggravating effect on CWP. One woman described this as:

“… it started sometime in my 40s because then I identified myself with older colleagues who were in pain then … I told them that, aha, now it starts on me, and then it was probably a knee or something that wasn’t working properly.” [1]

Insomnia and being stiff in the mornings were also experienced as having negative effects on CWP in forms of being triggering, aggravating, and consolidating. Other diseases, such as inflammations, psoriasis, scoliosis, and infections also had triggering or aggravating effects on CWP. One such experience was that CWP had emerged after an allergic reaction to medicine for another disease. One woman stated:

“… I had had a cold for a longer period, so I suppose they think that is what triggered it” [9].

## Discussion

In this study, a selection of participants was made in order to capture the story told by individuals who had gone from no chronic pain, or regional pain into CWP during their adult life. The women included described their journey in terms of triggering, aggravating, and consolidating factors, including both physical, psychological, and social aspects. The factors they capture in their description of the journey from the onset of CWP, towards living a life with persistent CWP is much in line with the risk factors identified in prospective cohort studies [13, 15]. Our findings further show that the women described their journey to CWP in accordance with the biopsycho-ecological model, which is an expanded version of the biomedical model and the biopsychosocial model [21]. Even though the biopsychosocial model is the most accepted approach related to the etiology of CWP [6, 7], it is insufficient in terms of involving all environmental aspects. The biopsycho-ecological model, on the other hand, includes both physical and social environments, as well as biological, psychological, and behavioral elements [21].

### Environmental circumstances

The results concerning the environmental circumstances entail that there are factors that cannot be affected by any person, such as the weather, while unmanageable work-related demands and a lack of social support can be influenced by others. Thus, interventions supporting women managing the complexity of living with CWP could be essential.

With regard to unmanageable work-related demands, previous research suggests that practitioners need to design effective workplace interventions to enable individuals to effectively manage pain and their work without serious implications as well as to prevent disability and promote health among workers with chronic pain [22]. Interventions of this type directed at women with CWP may benefit from considering several dimensions of work-related demands, as the results demonstrate the individual nature of the pain journey.

In terms of the women’s experiences of lack of support and distrust from, especially, the health care sector, this is in line with what was seen by Sluka et al. who also state that patients in the worst-case scenarios are told that their illness is only “in their head” and that there is nothing wrong with them, thus leading to them not being offered any treatment at all [23]. Goffman’s theory about stigmatization can be relevant in this context. Goffman suggests that a person is stigmatized when he or she deviates from specific expectations in an unfavorable way. This means that people with certain characteristics, ethnicities, and somatic dysfunctions who are considered undesirable by society are particularly exposed to negative responses from others. This can, for example, lead to inadequate treatment and increased distress and anxiety [24] and thus be an explanation to the experienced lack of social support. Furthermore, the experienced negative effects on the women’s pain journey due to lack of social support is in line with previous research, which suggests that identification and understanding of the illness believes among persons with CWP may reduce their suffering if they are considered in rehabilitation programs and in the development of interventions focused on promoting health among persons with CWP. Constraining illness believes, with special regards to a high number of physical or mental symptoms, beliefs of negative consequences or the illness affecting them emotionally, have namely been related to worse health status [25].

### Lifestyle

The women’s experiences regarding the impact of the lifestyle on their CWP entail that they could to some extent take control over the pain journey themselves, but that they had different capabilities for doing so. The women’s lifestyle was dependent on how they adjusted to the pain, where some perceived that they were more afraid of the pain than others. This can be related to catastrophic thinking about pain and fear avoidance, which have previously been shown to be important mechanisms that could help to explain why CWP can lead to reduced health-related quality of life [26]. People with chronic pain have shown greater catastrophic thinking about pain compared to those with acute pain [27]. If pain is perceived as threatening it can also lead to more severe symptoms, such as increased disability and depression. Fear of pain can many times have a greater impact than the degree of the initial injury itself, and patients who have less knowledge of pain, report more fear avoidance and higher levels of perceived disability because of pain [28]. It is, however, important to also consider the women in this study who ignored their condition and continued with activities that affected their pain negatively. This was similarly observed in a study about the life adjustment process in chronic pain by Gullacksen & Lidbeck, where some of the participants tried to convince themselves and others, for as long as they could, that they did not feel ill [29]. This study thus shows that denial as well as fear avoidance can have a negative impact on the journey to CWP.

### Personal attributes

The results concerning personal attributes further indicate that there are factors that both can and cannot be controlled. We thus maintain that it is important for people with CWP to learn about which factors they can control and how to do it so that they can identify ways to live with the pain and feel that they have as much control over their situation as possible. Prioritizing personal strength and focusing on what is pleasurable in life, instead of situations where the pain dominates, can thus be of importance here. This can be referred to as empowerment, which has been highlighted as a coping mechanism for chronic pain in other studies [30, 31].

Finally, the women in this study used different frameworks when explaining their pain journey in terms of triggering, aggravating, and consolidating factors. This is in line with what was found in the study by Richardson et al. in terms of biographical disruption, where the participants used frameworks to give meaning to their CWP, including triggering incidents and/or predisposing factors [9]. It is thus possible that the way the women in this study made sense of their illness in the context of their lives is partly a consequence of internalization of an attributed identity, which to some extent in return could be a consequence of stigmatization, since it can impair normal identity [24]. Listening to and believing the person who talks about his or her pain can thus have an important impact on the journey to CWP. This can be related to person-centered care, which encompasses acknowledging the patients’ perspectives of illness, needs, and preferences, which has been highlighted by other researchers as a central approach when meeting people with chronic pain [32].

In addition, a previous study found that patients treated for CWP are likely to favor physical rather than psychological interventions, although a psychological intervention had a similar effect [8]. A patient’s perception of the underlying mechanisms of their CWP is thus of importance for cost-effective care. The results from our study show that the women who had experienced this journey to CWP were aware of the complexity behind the cause, and the progression of regional pain to developing CWP. To acknowledge this complex interplay between physical, psychological, and social as factors of importance for their pain development ought to be of importance for compliance in interventions including a psychological approach.

### Strengths and limitations

The trustworthiness of this qualitative study can be based on its *credibility*, *dependability,* and *transferability*. Credibility relies on how well the data and processes of analysis address the intended focus of the research, where it is important to find participants who have experiences and can talk about the phenomenon [18, 20]. A strength of this study was the selection of participants including individuals who had been followed for 21 years, which introduced the possibility to identify people who we could follow regarding their CWP status over time. The inclusion criteria were to have developed CWP at some point between 1995, and 2016, enabling this study to capture the experience from developing CWP during adulthood. To only include women was a decision made upon the possibility that there may be a gender difference in the pain journey to CWP, and is a decision that can be considered both as a strength and a limitation. The decision was made although the research investigating gender-specific risk factors for the development of persistent CWP over a life span is very sparse. This study does not imply that there is likely a difference in the experience of living with CWP between men and women, however, there might be a difference in how men and women in general experience their journey to CWP. It is well known from previous studies that the prevalence of CWP is higher among women and that women are at increased risk for persistent CWP [13–15], which justify this decision to analyze experiences from men and women separately. This study does, however, highlight the importance of conductance of a similar study including only men. Dependability refers to the degree to which data changes over time and alterations made in the researchers´ decisions during the process of the analysis [20]. The researchers´ pre-understanding was considered in order to increase the dependability in this study [18]. All the authors had a background in the health and medical field, two of whom (IL, KA) had a clinical background, thus increasing the chances to understand the women and their experiences. On the other hand, there is a risk that the authors´ pre-understanding has led the result in a certain direction [18], and to minimize this risk all the authors were included in the analysis [18]. All subcategories and categories were discussed back and forth until a consensus among the authors was achieved. Transferability concerns the extent to which the findings are transferable to other contexts, and it is important to give an accurate and rich description of the method to increase a study’s transferability [18, 20]. A description of the method has been presented as clearly as possible, and the result is thus deemed to be transferrable to a wider population with other chronic pain conditions.

## Conclusions

The women experienced that environmental circumstances, lifestyle, and personal attributes affected their journey to CWP. The impact of these factors on the pain journey varied among women. These findings show that women are very conscious of their condition and can describe the broad context of how CWP is triggered, aggravated, and consolidated. This study thus confirms the complexity of individual pain progress and highlights the individual’s awareness of this complexity, which is important to consider when introducing interventions, and when expecting compliance to interventions. Furthermore, the implications of this study are that there is still work to be done when it comes to which preventive actions can be taken in society, such as improving work-related conditions and attitudes within health care services in relation to people with CWP.

## Data Availability

The datasets used and/or analyzed during the current study are available from the corresponding author on reasonable request.

## Abbreviation

CWP: Chronic widespread pain
